# Estradiol–Testosterone Imbalance Is Associated with Erectile Dysfunction in Patients with Klinefelter Syndrome

**DOI:** 10.3390/jcm10112319

**Published:** 2021-05-26

**Authors:** Maurizio De Rocco Ponce, Riccardo Selice, Antonella Di Mambro, Luca De Toni, Carlo Foresta, Andrea Garolla

**Affiliations:** 1 Andrology Department, Fundació Puigvert, Instituto de Investigaciones Biomédicas Sant Pau (IIB-Sant Pau), Departamento de Medicina, Universitat Autónoma de Barcelona, 08041 Barcelona, Spain; 2 Unit of Andrology and Reproductive Medicine, Department of Medicine, University of Padova, Via Giustiniani 2, 35128 Padova, Italy; riccardo.selice@aopd.veneto.it (R.S.); antonella.dimambro@aopd.veneto.it (A.D.M.); detoni.luca@gmail.com (L.D.T.); carlo.foresta@unipd.it (C.F.); andrea.garolla@unipd.it (A.G.)

**Keywords:** erectile dysfunction, Klinefelter syndrome, testosterone, estradiol, hypogonadism

## Abstract

Erectile dysfunction (ED) is a frequent sexual disorder in adult men. Klinefelter syndrome (KS) is the most common sex chromosomal disorder and a frequent cause of male hypogonadism. Psychological and cognitive aspects are quite typical in KS and have been linked to ED, while the role of testosterone (T) levels in sexual function of KS subjects has not been fully elucidated. The purpose of the present study is to investigate the role of hormonal disturbances in erectile function of subjects with KS. We conducted a retrospective study involving 52 Klinefelter patients newly diagnosed who never received androgen replacing therapy. All the subjects underwent medical history, accurate physical examination, and blood tests. The International Index of Erectile Function questionnaire (IIEF-EF) score correlated negatively with estradiol/testosterone ratio (E2/T); this correlation remained statistically significant after correction for age (ρ −0.320 *p* = 0.018). A multiple linear regression analysis identified age and E2/T as the main predictors of IIEF-EF score (R2 0.169 F = 3.848 *p* = 0.008). Our findings corroborate previous KS data obtained in the general population showing an association between higher E2/T ratio and impaired erectile function. Larger studies are required to better elucidate the pathophysiology of ED in patients with KS.

## 1. Introduction

Erectile dysfunction (ED) is the most common sexual disorder in adult men along with premature ejaculation [1]. ED is defined as the persistent inability to obtain or maintain a penile erection firm enough for satisfactory sexual intercourse [2]. An incidence of 25–30 new cases per 1000 people has been reported, and it is expected that by 2025 there will be a prevalence of 322 million people affected all over the world [3]. In the Massachusetts Male Aging Study, a global prevalence of 52% in patients between 40 and 70 years of age was reported. Moreover, the prevalence of ED increases with age, reaching 67% in men over 70 years [4]. Other major risk factors are smoking, sedentarism, and common chronic diseases such as hypertension, hypercholesterolemia, obesity, and diabetes mellitus [5]. Moreover, ED represents a symptom that can be related to different pathophysiologic processes involving vascular, neurological, or endocrinological impairment, among others. Moreover, psychological and relational disorders may also lead to ED. According to the main underlying condition, different subtypes of ED may be defined [5]. ED is named “vasculogenic” when it is related to impaired cavernous artery blood inflow or to venous outflow disorders. Vasculogenic ED is the most frequent type of ED in adult and older men (up to 70% of all cases) [6]. Recent guidelines on male sexual dysfunction suggest assessing the hormonal status during the diagnostic work-up of ED because hormonal disturbances can be involved in developing ED [7]. In particular, testosterone (T) deficiency (i.e., hypogonadism) is associated with several sexual symptoms including ED [8], and it can play an important role in erectile disorders [9]. Many studies have shown detrimental effects of hypogonadism on penile erectile tissue, while androgen replacement therapy can partly restore erectile function [10,11]. Interestingly, data from both animal and human studies showed a possible relationship between estrogens (E2) and erectile function [12,13,14,15,16]. Moreover, some authors hypothesized that ED could be related to the imbalance between T and E2 rather than to a direct effect of estrogens themselves [17]. However, the role of estrogens in this context is still debated.

Klinefelter syndrome (KS) has a prevalence of 1:600, and it is the most common sex chromosomal disorder. KS is also a frequent cause of male hypergonadotropic hypogonadism [18]. In fact, subjects with KS often present with hypergonadotropic hypogonadism, testicular hypotrophy, and infertility [19], along with a higher incidence of systemic diseases such as venous thromboembolism, diabetes, cardiovascular diseases, metabolic impairments, osteoporosis, and cancer [20,21,22,23]. Furthermore, psychological and cognitive aspects are quite typical in KS and have been extensively investigated [24]. In particular, some psychological disturbances have been linked to ED in KS, while the role of T levels and hypogonadism in sexual function of subjects with KS is not fully elucidated [25]. Moreover, sexual function in KS has not been studied in depth, and only a few studies have investigated the different roles of hormonal, neuropsychological, cognitive, and relational disturbances.

The purpose of the present study is to investigate the role of hormonal disturbances on the erectile function of subjects with KS. In particular, we explore the role of T, E2, and their imbalance in the physiopathology of ED among patients with KS.

## 2. Materials and Methods

### 2.1. Study Type and Subjects of the Study

The present is a retrospective study involving 52 patients with KS newly diagnosed at the Unit of Andrology and Reproductive Medicine of the University Hospital of Padua from January 2014 to December 2019. Eligible patients were sexually active subjects with KS and non-mosaic 47, XXY karyotype who had never received androgen replacing therapy (ART) at the time of evaluation. The study was approved by the Ethics Committee of the University Hospital of Padova (Protocol number 2357P), and each participant gave his written informed consent. We excluded patients with mosaicism or more than one supernumerary X chromosome. We also excluded patients with post-surgical ED, Peyronie’s disease, neoplastic history, end-stage renal or liver disease, neurological disease, any endocrine dysfunction different from hypogonadism, and subjects consuming any drug.

### 2.2. Clinical Assessment

All the subjects underwent an accurate physical examination with anthropometric measurements (weight, height, body mass index (BMI), waist circumference (WC), testicular volume by orchidometer comparison) and medical history collection including pubertal history, smoke habit, and alcohol misuse. All subjects completed the International Index of Erectile Function questionnaire for erectile function (IIEF-15) [26]. Erectile dysfunction was defined as an erectile function (EF) subdomain < 25 points. Blood tests included fasting plasma glucose, glycated hemoglobin (HbA1c), fasting insulin, Homeostatic Model Assessment of Insulin Resistance (HOMA-IR) calculation, serum lipid profile, hormone levels (luteinizing hormone (LH), follicle stimulating hormone (FSH), total testosterone (TT), free testosterone (FT), estradiol (E2), prolactin (PRL), and thyroid stimulating hormone (TSH)), and total prostate-specific antigen (PSA). Hypogonadism was defined as TT < 10.4 nmol/L [27]. Blood collection and pressure measurements were performed in fasting conditions and avoiding cigarette smoking for a minimum of 12 h. FSH, LH, TT, and E2 were evaluated by commercial electrochemiluminescence immunoassay (Elecsys 2010; Roche Diagnostics, Mannheim, Germany). Karyotype was determined after evaluation of at least 50 peripheral blood lymphocyte metaphases.

### 2.3. Data Analysis

Data are expressed as mean ± standard deviation for continuous variables or as N with percentage for categorical variables. The normal distribution of the variables was assessed by the Kolmogorov–Smirnov test, and not all variables were normally distributed. For correlation, a Spearman’s test was performed. For comparison of continuous variables between groups a Mann–Whitney test was performed, while the Pearson X2 test or the exact Fisher test was performed for categorical variables. Multiple linear regression analyses were performed to test the effect of different parameters, whenever indicated. A *p* value < 0.05 was assumed as statistically significant. Statistical analysis was performed using SPSS statistics software for Windows (SPSS Inc., Chicago, IL, USA).

## 3. Results

Fifty-two patients with KS were enrolled. Patients’ ages ranged from 18 to 58 years (mean 31.2 ± 7.9 years). None of them had diabetes mellitus, 51.9% had dyslipidemia (i.e., total-cholesterol > 200 mg/dl or LDL-cholesterol > 130 mg/dl), 7.1% had hypertension, 60.3% were current smokers, and 53.4% had hypogonadism. The main characteristics of enrolled patients are presented in Table 1.

Twelve patients (21.1%) had ED according to their IIEF-EF score, while 46 (78.9%) had normal IIEF-EF scores (no-ED). Patients with ED were significantly older (36.8 ± 9.7 vs. 29.6 ± 6.7 years *p* = 0.020), with a higher prevalence of hypertension (25% vs. 2.3% *p* = 0.028) and higher triglycerides (183 ± 15 vs. 112 ± 14 mg/dL *p* = 0.006). Moreover, they presented lower TT (7.8 ± 5.5 vs. 10.7 ± 4.4 nmol/L *p* = 0.046) with higher estradiol/total testosterone ratio (E2/T: 17.9 ± 10.1 vs. 11.0 ± 6.2 *p* = 0.005). The characteristics of ED and no-ED patients are detailed in Table 2.

IIEF-EF score correlated positively with TT (ρ 0.303 *p* = 0.021) and negatively with age (ρ −0.333 *p* = 0.011) and E2/T (ρ −0.378 *p* = 0.003).

After correction for age, TT was no longer associated with IIEF-EF score. In contrast, E2/T remained statistically correlated with IIEF-EF score after correction for age (ρ −0.320 *p* = 0.018).

A multiple linear regression analysis identified age (β = 0.294 *p* = 0.033) and E2/T (β = 0.483 *p* = 0.0.18) as the main predictors of IIEF-EF score (R2 0.169 F = 3.848 *p* = 0.008).

**Figure 1:**
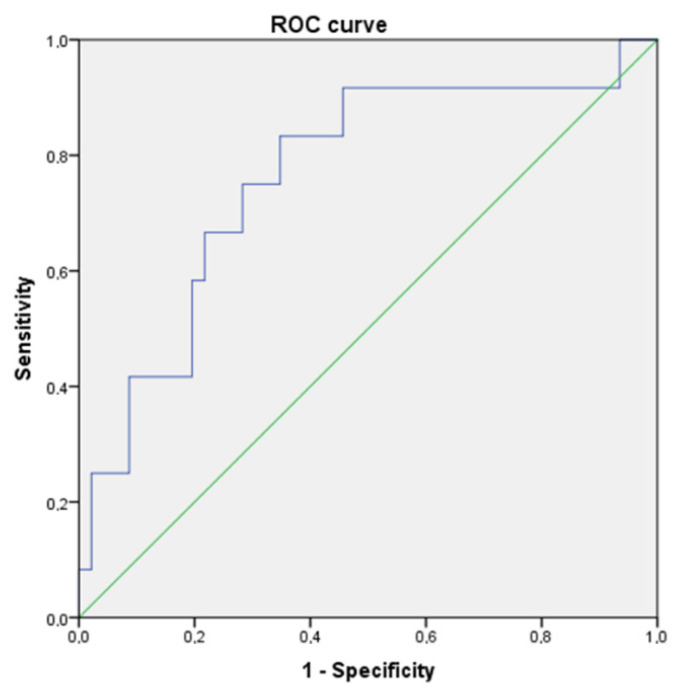
E2/T ROC (Receiver Operating Characteristic) curve for ED. AUC (Area Under Curve) = 0.763.

The E2/T ROC curve for ED showed an AUC of 0.763 (95% CI 0.604–0.921) with no clear cut-off. However, a E2/T threshold of 10.3 pmol/nmol would provide a sensitivity of 83% and specificity of 65% (Figure 1).

## 4. Discussion

Sexual dysfunction is considered a possible presentation for patients with KS [28]. It is believed that after the age of 25, about 70% of patients with KS complain about decreased libido and erectile dysfunction [29]. Yoshida found that 67.5% of subjects with KS have at least one sexual function disturbance [30]. In our study we found a prevalence of ED among patients with KS of 21.1%, which is comparable with previous literature regarding the prevalence of ED in KS [31].

It is well acknowledged that T plays a role in many aspects of normal sexual function such as desire, arousal, orgasm, and ejaculation. Therefore, ED in KS is commonly considered to be secondary to the hypogonadism usually present [32]. In fact, Corona et al. reported the association between KS and severe ED, hypoactive sexual desire, and premature and delayed ejaculation. Nevertheless, these association disappeared when compared with control subjects matched for age, smoking habit, and T, so the authors concluded that sexual dysfunction in KS is mainly due to hypogonadism [33]. On the other hand, other data showed that ED in KS is also linked to psychological disturbances, while erectile function seems to be less related to T levels and hypogonadism. Moreover, T replacement therapy is not able to improve erections [25]. Therefore, the exact role of T in the sexuality of KS patients is still debated.

In our study, even if patients with ED presented significantly lower TT levels, erectile function measured with the IIEF-EF questionnaire was not independently associated with TT after correction for confounding factors. In 2013, El-Sakka et al. suggested a possible role of E2 in ED. They performed a clinical study in 614 middle-aged subjects with ED and found a negative correlation between lower IIEF-5 score and both low TT with high E2 or normal/low TT and/or elevated E2 [13]. Actually, E2 receptors had been already demonstrated in both smooth muscle cells and endothelium of human corpus cavernosum [34]. After that, Vignozzi et al. showed in a rabbit model that metabolic syndrome-induced ED was more associated with higher E2 rather than with lower T levels [12]. Recently, Xu et al. demonstrated that E2 was able to reduce erectile function assessed by nocturnal penile tumescence rigidity test (Rigiscan) in 135 non-diabetic men [15]. More recently, Chen et al. compared 195 eugonadic subjects with organic or psychogenic ED and 52 healthy men by the IIEF questionnaire, Rigiscan, and penile color-doppler ultrasound (PCDU). Their data showed a correlation between higher E2 levels and organic ED, defined as a worse penile rigidity in the Rigiscan. We confirmed these findings in work on patients with type 2 diabetes mellitus (T2DM), where we found a correlation between E2 levels and IIEF-5 scores [35]. In the present study E2 levels are not independently correlated with erectile function. However, the underlying mechanisms in KS may be partly different from those in T2DM. For example, although the expression of aromatase has been reported to be four times higher in testis of men with KS [36], in our study this did not translate into a condition of peripheric hyperestrogenism as in the patients with diabetes mellitus. A possible explanation may be the concomitant normal–low testosterone levels with low aromatase substrate availability.

Other studies evidenced a relative hyperestrogenism (i.e., increased E2/T) in men with KS [37]. Interestingly, in the present study the E2/T ratio emerged as independently correlated with IIEF-EF score, and this correlation remained statistically significant after correction for age, TT, and E2. Moreover, the multiple linear regression analysis supported the value of E2/T in relation to ED, as age and E2/T were the main predictors of IIEF-EF score. The impact of age on the development of ED is not surprising, since age is a known risk factor for ED, and in the general population, the incidence of ED increases with age [4]. In our study also, patients with ED were significantly older. From this point of view, we confirmed in subjects with KS the importance of ageing as risk factor in the natural history of ED. On the other hand, why E2/T is a better predictor rather than TT or E2 levels alone is an intriguing question. A possible explanation is that in a context of normal–low levels of TT, the E2/T ratio is a more sensitive parameter of hormonal balance in relation to erectile function than TT or E2 levels alone. This hypothesis could be supported by some previous works. For instance, in 2016, Wu et al. performed a study about sexual dysfunction in 878 men including 292 patients with ED and 347 controls without ED. In that study, they found that ED patients showed higher E2 levels in comparison with controls, with no statistical difference in testosterone concentrations. Importantly, the E2/T was also higher than in normal control subjects [14].

As regards the pathophysiology underlying this association between E2/T and erectile function in patients with KS, we can only speculate as we lack other data such as PCDU assessment to support any hypothesis. However, extending the analysis to data from the other IIEF-15 domains, we found a significant negative association between E2/T with the IIEF-15 desire domain (ρ −0.369 *p* = 0.005). Moreover, KS subjects with ED presented significantly worse scores in the IIEF-15 sexual desire subdomain, and significantly worse intercourse and overall satisfaction scores, while the orgasmic function subdomain was not statistically different (Table 2). This means that erectile function and desire are covariates negatively associated with E2/T and that low desire and ED may influence each other in patients with KS. In fact, these results are similar to those by El Bardisi et al., who found a significantly higher incidence of low libido in KS patients vs. controls (54.7% vs. 17.3%, respectively) despite normal testosterone levels [31].

Taken altogether, our data suggest a negative effect of higher E2/T ratio on erectile function. However, the exact mechanisms underlying this association is unclear, as low sexual desire may play a role, while we do not have data about penile vascular function.

To the best of our knowledge, this is the first study investigating the role of estradiol and testosterone imbalance in relation to sexual function in patients with KS. The major limitation of this study is the small sample size that may not be sufficient to show consistent or further correlations. Moreover, concentrations of albumin and sex hormone binding globulin were not available; thus, possible fluctuations of the bioactive amount of E2 and T could not be assessed. We also lacked PCDU assessment as an objective assessment of penile vascular function. Finally, data from a specific psychological assessment were not available; therefore, we could not properly evaluate the role of any psychological alteration in the pathophysiology of ED in our patients.

## 5. Conclusions

Our findings confirm for the first time in patients with KS data previously obtained in the general population showing an association between higher E2/T and impaired erectile function. Low sexual desire may play a role, but the exact mechanisms underlying this association remain unclear. Further studies with larger samples are required to better elucidate the pathophysiology of the interaction between estrogen–testosterone imbalance and erectile function, both in patients with KS and in the general population.

## Figures and Tables

**Table 1:** General characteristics of the study subjects.

General Characteristics of the Study Subjects			
Age (years)	31.2 ± 7.9	TSH (mU/L)	1.80 ± 0.97
BMI (kg/m^2^)	26.2 ± 5.3	LH (UI/L)	21.7 ± 6.1
Waist circumference (cm)	99 ± 16	FSH (IU/L)	35.2 ± 12.2
Glycemia (mg/dL)	82 ± 15	Total testosterone (nmol/L)	10.14 ± 4.79
HbA1c (%)	5.4 ± 0.4	Hypogonadism (%)	31 (53.4)
HOMA-IR	2.3 ± 1.8	Free testosterone (nmol/L)	0.20 ± 0.09
Total cholesterol (mg/dL)	201 ± 46	Estradiol (pmolLl)	99.2 ± 34.6
HDL-cholesterol (mg/dL)	48 ± 11	Prolactin (ng/mL)	10.7 ± 6.3
Triglycerides (mg/dL)	152 ± 238	PSA (ng/mL)	0.59 ± 0.36
LDL-cholesterol (mg/dL)	126 ± 35	Total testicular volume (mL)	3.8 ± 1.2
Smoke habit (%)	35 (60.3)	IIEF-EF (score)	26.7 ± 6.6
Hypertension (%)	4 (7.1)	Erectile dysfunction (%)	12 (21.1)

BMI: body mass index, HbA1c: glycated hemoglobin, HOMA-IR: Homeostatic Model Assessment of Insulin Resistance; TSH: thyroid stimulating hormone, LH: luteinizing hormone, FSH: follicle stimulating hormone. Continuous variables are expressed as mean ± standard deviation. Categorical variables are expressed as *n* (%).

**Table 2:** Comparison between patients with ED and without ED.

	ED (*n* = 12)	No-ED (*n* = 46)	*p*-Value
Age (years)	36.8 ± 9.7	29.6 ± 6.7	0.020
BMI (kg/m^2^)	27.5 ± 1.3	25.9 ± 0.8	0.313
Waist circumference (cm)	100 ± 3	98 ± 2	0.477
Glycemia (mg/dL)	88 ± 8	81 ± 1	0.751
HbA1c (%)	5.5 ± 0.2	5.3 ± 0.1	0.615
HOMA-IR	2.48 ± 0.62	2.36 ± 0.26	0.953
Total cholesterol (mg/dL)	216 ± 19	197 ± 6	0.508
HDL-cholesterol (mg/dL)	43 ± 3	49 ± 2	0.098
Triglycerides (mg/dL)	183 ± 15	112 ± 14	0.006
LDL-cholesterol (mg/dL)	137 ± 9	124 ± 5	0.245
Smoke habit (%)	7 (58.3)	28 (60.9)	0.873
Hypertension (%)	3 (25.0)	1 (2.3)	0.028
TSH (mU/L)	1.90 ± 0.19	1.78 ± 0.15	0.253
LH (UI/L)	20.1 ± 1.9	22.1 ± 0.9	0.472
FSH (IU/L)	30.8 ± 2.7	36.9 ± 1.8	0.182
Total testosterone (nmol/L)	7.8 ± 5.5	10.7 ± 4.4	0.046
Hypogonadism (%)	8 (66.7)	23 (50.0)	0.303
Free testosterone (nmol/L)	0.16 ± 0.03	0.21 ± 0.01	0.201
Estradiol (pmol/L)	95 ± 10	100 ± 5	0.818
E2/T (pmol/nmol)	17.9 ± 10.1	11.0 ± 6.2	0.005
Prolactin (ng/mL)	13.2 ± 3.4	10.0 ± 0.6	0.810
PSA (ng/mL)	0.49 ± 0.08	0.62 ± 0.05	0.328
IIEF-EF (score)	15.7 ± 7.7	29.5 ± 1.0	<0.001
IIEF-OD (score)	8.75 ± 2.3	9.8 ± 0.5	0.135
IIEF-SD (score)	5.7 ± 1.7	7.7 ± 1.6	<0.001
IIEF-IS (score)	6.8 ± 4.6	12.0 ± 3.1	0.003
IIEF-OS (score)	6.2 ± 2.4	8.1 ± 1.2	0.042

Characteristics of patients with erectile dysfunction (ED) and without erectile dysfunction (No-ED). BMI: body mass index, HbA1c: glycated hemoglobin, HOMA-IR: Homeostatic Model Assessment of Insulin Resistance; TSH: thyroid stimulating hormone, LH: luteinizing hormone, FSH: follicle stimulating hormone. IIEF-EF: IIEF-15 erectile function domain, IIEF-OD: IIEF-15 orgasmic function domain, IIEF-SD: IIEF-15 sexual desire domain, IIEF-IS: IIEF-15 intercourse satisfaction domain, IIEF-OS: IIEF-15 overall satisfaction domain. Continuous variables are expressed as mean ± standard deviation. Categorical variables are expressed as *n* (%).

## Data Availability

Not applicable.
